# Advancements in Biochar as a Sustainable Adsorbent for Water Pollution Mitigation

**DOI:** 10.1002/advs.202410383

**Published:** 2025-04-17

**Authors:** Devika Laishram, Su‐Bin Kim, Seul‐Yi Lee, Soo‐Jin Park

**Affiliations:** ^1^ Department of Mechanical Engineering Kyung Hee University Yongin 17104 Republic of Korea; ^2^ Department of Advanced Materials Engineering for Information and Electronics Kyung Hee University Yongin 17104 Republic of Korea

**Keywords:** biochar, biomass, machine learning, wastewater treatment

## Abstract

Biochar, a carbon‐rich material produced from the partial combustion of biomass wastes is often termed “black gold” for its potential in water pollution mitigation and carbon sequestration. By customizing biomass feedstock and optimizing preparation strategies, biochar can be engineered with specific physicochemical properties to enhance its effectiveness in removing contaminants from wastewater. Recent studies demonstrate that biochar can achieve > 90% removal efficiency for heavy metals such as lead and cadmium, > 85% adsorption capacity for organic pollutants such as dyes and phenols, and > 80% reduction in microplastics and nanoplastics. This review explores recent advancements in biochar preparation technologies, such as pyrolysis, carbonization, gasification, torrefaction, and rectification, along with physical, chemical, and biological modifications that are crucial for efficient pollutant removal. The core of this review focuses on biochar's applications in removing a wide range of pollutants from wastewater, detailing mechanisms for organic pollutants, inorganic salts, pharmaceutical contaminants, microplastics, nanoplastics, and volatile organic compounds. In addition, the review introduces machine learning as a key technique for optimizing biochar production and functionality, showcasing its potential in advancing biochar technology. The conclusion provides a comprehensive outlook on biochar's future, emphasizing ongoing research and its role in sustainable environmental management.

## Graphical abstract

Biochar, obtained through pyrolysis of organic waste, serves as a sustainable solution for wastewater treatment due to its adaptability and low‐cost nature. This review comprehensively examines recent advancements in biochar production, functional modifications, and applications, highlighting the integration of machine learning and artificial Intelligence to optimize biochar properties for efficient removal of contaminants such as pharmaceuticals, microplastics, and organic/inorganic pollutants.

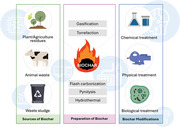

## 1 Introduction

Anthropogenic wastewater production and water body contamination have severely impacted public health and ecosystems, leading to the significant degradation of aquatic habitats and loss of biodiversity. Contaminated drinking water sources contribute to the proliferation of waterborne diseases, posing serious health risks to humans and animals. A report by the World Health Organization (WHO) stated that there are 785 million people that do not have access to potable water in 2019, and by 2050, 50% of the world's population will not have access to clean drinking water.^[^ ^1^ ^]^ Globally, ≈1000 km^3^ of wastewater is generated annually, with nearly 30% from municipal and over 60% from industrial sources.^[^ ^2^ ^]^ Major sources of water pollution include industrial discharges, agricultural runoff, sewage, oil spills, and plastic waste. Major constituents of these pollutants include toxic and harmful substances such as perfluorinated compounds, personal care products (PCCPs), cosmetic products, persistent organic pollutants (POPs), steroids, and endocrine disrupting hormones, to name a few. The increasing presence of pharmaceuticals, microplastics, heavy metals, and other emerging contaminants in water bodies exacerbates the problem, highlighting the urgent need for effective pollution control and sustainable water management practices. For example, in a recent article, Batool et al. reported the use of nanocomposite based upon a carbon nanohorn (CNH)‐modified BiSbS_3_/BiSbO_4_ to treat pentachlorophenol (5‐CP), which is an endocrine disruptor and can even cause hormone‐induced cancers.^[^ ^3^ ^]^

When pollutants are mixed with the surface water discharge, wastewater contamination is aggravated and rises substantially from the parent source of water. In addition, there are emerging pollutants such as nanoparticle and microplastic pollutants with very little understanding and research especially in developing countries due to lack of resources and technology. For example, the presence of pollutants in trace amount requires highly sensitive analytical instruments that can perform measurements in nanograms or milligrams per liter.^[^ ^4^ ^]^ The long‐term effect on human and environment health is yet to be established; and therefore, poses great risk to the humans, animals, and environment exposed to such conditions.

A cost‐effective and simple process; yet effective, is most attractive for eliminating the pollutants. There are various techniques reported for the removal of these pollutants from water, which include anodization along with biosorption and chemical oxidation, reverse osmosis, advanced oxidation process, and biodegradation. However, these processes are energy intensive and face other problems such as fouling, disposal of the brine, and inefficiency in removal of the emerging pollutants.^[^ ^5^ ^]^ In this regard, adsorption processes are highly desirable as it is effective and allows for large scale treatment of the effluents. In general, the materials for adsorption can be sourced naturally but its function is limited by its capacity for adsorption, lack of functional group, and recyclability. Most commonly used materials for adsorption include activated carbon, which is particularly attractive owing to its capacity to remove a large variety of pollutants including organic and inorganic contaminants, dyes, metals, ions, chlorinated and unchlorinated phenolic compounds, and other pollutants such as pesticides.^[^ ^4^ ^]^ Garg and co‐workers published a recent review on the use of aerogels as an agent for treatment of wastewater.^[^ ^6^ ^]^ Aerogels are porous, light, and biocompatible and have been explored for various other applications. While advantageous, using aerogels also suffers from certain drawbacks such as reduced desorption rate due to strong electrostatic force creating chemisorption, complex, lengthy fabrication process, and so on. In addition, the material used for adsorption heavily relies upon the kind and nature of the targeted pollutants. Traditional wastewater treatment methods often struggle to address the complex and diverse nature of modern pollutants. Further, these conventional approaches are often energy‐intensive and contribute to greenhouse gas emissions, thereby exacerbating environmental issues. The United Nations Sustainable Development Goals (SDGs) for 2030 emphasize the urgent need for sustainable water treatment technologies to promote societal and economic development.^[^ ^7^ ^]^ One promising solution aligning with these goals is the use of biochar due to its multifaceted environmental benefits and potential for sustainable development. Recent studies demonstrate that biochar can achieve > 90% removal efficiency for heavy metals such as lead and cadmium, > 85% adsorption capacity for organic pollutants such as dyes and phenols, and > 80% reduction in microplastics and nanoplastics.^[^ ^8^ ^]^ Machine learning has already been reported for optimizing biochar production and functionality, highlighting its potential improvement in efficient biochar use by 12.7%.^[^ ^9^ ^]^ In addition, biochar has the potential to sequester up to 1 to 35 gigatons of CO₂ annually and contribute significantly to global climate goals.^[^ ^10^ ^]^

Biochar is a carbon‐rich material produced through the thermal decomposition of biomass via pyrolysis, a process involving heating biomass at high temperatures in the absence of oxygen. Biomass holds significant potential as a natural macromolecular resource due to its widespread availability and cost‐effectiveness. It can be derived from various organic sources, including plant‐derived lignocellulosic components such as lignin, cellulose, and hemicellulose,^[^ ^11^ ^]^ as well as non‐lignocellulosic elements such as proteins, lipids, minerals, sugars, and other derivatives from plants, animals, and microorganisms.^[^ ^12^ ^]^ In addition, biomass can be sourced from municipal waste, agricultural waste (e.g., straws, crop residues, woodchips, and livestock), and industrial organic wastes from textile and food processing residues.^[^ ^13^ ^]^ Common methods for biomass treatment include landfilling, conversion to combustible fuels such as biogas using microorganisms or enzymes, and thermochemical conversion at high temperatures to produce methane, hydrogen, and other gases.^[^ ^14^ ^]^ Despite its potential, direct burning of biomass, particularly agricultural residues such as straws, is prevalent. This practice releases CO_2_ and soot pollutants, including PM2.5 particles, metal oxides, oxynitrides, methylbenzene, and aldehydes, all contributing to global warming. In contrast, biochar production and utilization offer a more sustainable and environmentally friendly approach. Biochar production sequesters carbon in a stable form, reducing greenhouse gas emissions. For example, its application in soil enhances water retention, nutrient availability, and microbial activity, leading to enhanced agricultural productivity and reduced reliance on chemical fertilizers.^[^ ^15^ ^]^ Consequently, biochar helps mitigate the adverse environmental impacts associated with traditional biomass use.

Biochar is a promising candidate for pollutant removal from wastewater, carbon sequestration, and greenhouse gas mitigation due to its highly porous structure and large surface area.^[^ ^16^ ^]^ In addition, biochar possesses attributes such as hydrophobicity, long‐term stability, and a distinct chemical composition. Its stable carbon matrix allows for effective carbon sequestration and pollutant removal from the environment over extended periods.^[^ ^17^ ^]^ The physicochemical properties of biochar can be further fine‐tuned to function as a multifaceted material, capable of not only removing pollutants but also facilitating their mineralization by acting as a catalyst or reducing them to less toxic, easily degradable by‐products. This prevents the transfer of pollutants from one medium to another, as observed in processes such as membrane separation.^[^ ^18^ ^]^

Biochar, derived primarily from plant and animal organic waste, provides a sustainable and environmentally friendly option for treating wastewater.^[^ ^8b^ ^]^ In the process of eliminating contaminants, biochar effectively eliminates toxins; while, preserving nutrients and other valuable organic matter that can be further utilized in agriculture or energy generation. Therefore, biochar derived from biomass aligns with the principles of a circular economy, promoting resource efficiency, waste reduction, and environmental sustainability. The circular economy aims to create a sustainable economic model by maximizing resource efficiency and addressing environmental concerns through the principles of reuse, reduction, and recycling to minimize waste production and optimize resource utilization.^[^ ^19^ ^]^

**Figure 1:**
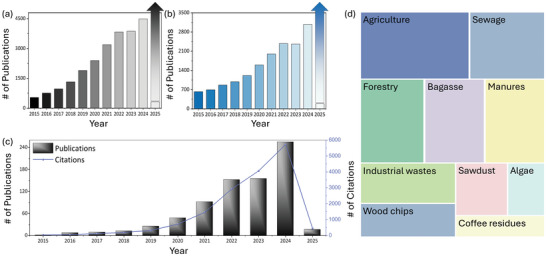
a) Number of publications in the environmental field based on biochar, b) number of publications on water purification in environmental field research, c) number of publications and citations in the environmental field using biochar for water purification, and d) most widely used biosource for biochar preparation (All publications and citation data were obtained from Web of Science, 2015–2025).

With the increasing interest in utilizing biochar for environmental applications, the number of publications on the role of various biomass‐derived biochars in removing pollutants from wastewater has also increased over the past decade, as shown in **Figure** **1**. This significant upward trend reflects the expanding research efforts and increasing interest in biochar material for various environmental applications. The increase in publications suggests advancements in understanding the unique properties of biochar, such as biocompatibility, textural characteristics, and environmental benefits. This trend also indicates broader acceptance and recognition of biochar as a valuable material for developing innovative and sustainable solutions in water remediation. Despite the growing interest in biochar's potential, there is a lack of detailed synthesis on the mechanisms of pollutant removal and the role of emerging technologies such as machine learning in optimizing biochar functionality. By filling this gap, the review aims to provide a valuable resource for researchers and practitioners seeking to enhance the efficacy of biochar in environmental management.

This review explores recent advancements in the application of biochar for wastewater treatment, focusing on pollutant removal mechanisms. It begins by discussing biochar preparation methods and subsequent modifications. It then explores the effectiveness of biochar in removing a wide range of pollutants from wastewater, such as organic pollutants, inorganic salts, pharmaceutical contaminants, microplastics, nanoplastics, and volatile organic compounds (VOCs). Further, it investigates the role of machine learning in optimizing biochar production and functionality, highlighting its potential to advance biochar technology. Biochar is sourced from agricultural, forestry, and industrial waste, utilizing biomass that would otherwise decay. The resulting biochar plays a crucial role in managing pollutants and contaminants, as detailed in the review. Overall, biochar production and usage can be considered as a closed‐loop waste management system that promotes the concept of circular economy. Adopting biochar can enhance sustainability both ecologically and economically through sequestering carbon by utilizing waste and remediating pollutants.

## 2 Preparation of Biochar

**Figure 2:**
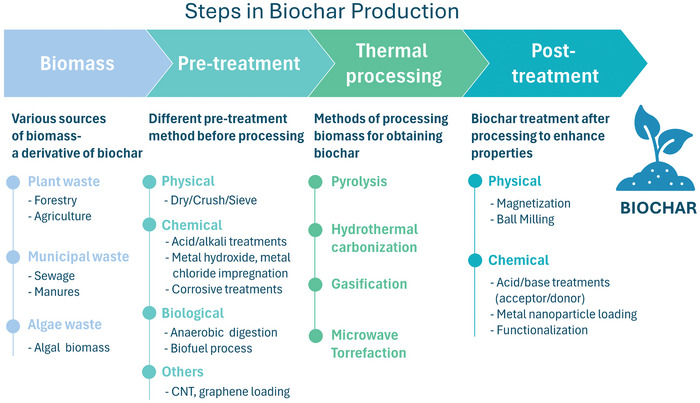
Overview of the steps and processes involved in biochar production.

The production of biochar involves several steps, as shown in **Figure** **2**. The process begins with the collection of biomass feedstock, which may come from agricultural residues, organic products from plants and animals, and municipal and industrial waste. This is followed by pretreatment, which includes physical, chemical, and biological methods.

### 2.1 Pretreatment

Physical pretreatment typically involves drying the biomass, followed by crushing or shredding, sieving, and washing. The specific steps may vary depending on the type and nature of the feedstock, such as its moisture content, particularly for plant‐derived biomass. Chemical pretreatment typically involves using a chemical activator to treat or impregnate the biomass, facilitating dehydration, enhancing the carbonization process, and preventing the formation of by‐products such as VOCs and tar. This pretreatment method also enables the incorporation of functional agents into the feedstock. For instance, acids, alkalis, and other corrosive chemicals are used in this step to enhance the pore structure of the resulting biochar.^[^ ^20^ ^]^ For example, the use of acidic chemical agents such as phosphoric acid (H_3_PO_4_) can break bonds without affecting the internal pore structure.^[^ ^21^ ^]^ Fierro et al. treated rice straw biomass with H_3_PO_4_ and observed that a H_3_PO_4_: biomass ratio of 1 increased the yield by 10%.^[^ ^22^ ^]^ Liu et al. used a mixture of H_3_PO_4_ and sulfuric acid (H_2_SO_4_) to treat walnut shells, introducing phosphorus atoms into the carbon matrix and causing sulfonation.^[^ ^23^ ^]^ Biochar derived from tea waste was treated with chloric acid (HCl), nitric acid (HNO_3_), and H_2_SO_4_; and Peiris et al. noted that the HCl treatment facilitated nucleophilic substitution, while, the HNO_3_ treatment led to the formation of nitrogen‐containing organic groups via electrophilic substitution. All acids were found to convert mesoporous structures to micropores.^[^ ^24^ ^]^ In addition, acid‐treated biochar has shown an increase in oxygen‐containing functional groups such as carboxyl groups.

Biochar activated with alkali agents such as potassium hydroxide (KOH) and ammonium hydroxide (NH_4_OH) typically exhibited high capacitance, making them suitable for electrochemical studies and energy storage applications.^[^ ^25^ ^]^ Modification using alkali agents enhanced the specific surface area of biochar. For example, Liu et al. reported an increase in surface area and total pore volume from 12 to 107 m^2^ g^−1^ and from 0.004 to 0.006 cm^3^ g^−1^, respectively.^[^ ^23^ ^]^ Further, El‐Nemr et al. found that the NH_4_OH treatment enriched the biochar structure with nitrogen. These treatments not only introduced functional groups but also enhanced the textural properties of biochar.^[^ ^26^ ^]^ Metal salts, such as chlorides and phosphates, significantly impact the texture and surface chemistry of biochar. For example, Zhang et al. noted that pretreatment with tripotassium phosphate (K_3_PO_4_) resulted in diphosphate (P_2_O_7_)‐enriched biochar, leading to the breakdown of polymers in the biomass. This pretreatment induced structural and textural changes, such as the formation of oxygen‐containing groups, and improved stability due to the “pre‐aging” effect.^[^ ^27^ ^]^

Biomass can be suspended in chemical solutions of metal chlorides, such as FeCl_3_, AlCl_3_, or MgCl_2_, to form metal oxyhydroxide nanocomposites.^[^ ^28^ ^]^ Metal chlorides have been investigated for similar purposes as alkali agents; however, Brenton et al. found that chlorides may clog pores, thereby reducing surface area and total pore volume.^[^ ^29^ ^]^ Cost‐effective chemical agents, such as hydrogen peroxide, have also been utilized, resulting in the generation of hydroxyl and carboxylic groups and an increase in the atomic O/C ratio.^[^ ^30^ ^]^ Recent studies have explored multiple modifications. For example, Yang et al. utilized potassium permanganate (KMnO_4_) and aluminum chloride hexahydrate (AlCl_3_·6H_2_O) in biochar formation, leading to a significant increase in the specific surface area and pore volume of 1173.3 m^2^ g^−1^ and 0.54 cm^3^ g^−1^, respectively.^[^ ^31^ ^]^ Organic‐based chemicals, such as chitosan, polyethyleneimine, and vitamin B6, have been employed, leading to changes in surface chemistry with the introduction of new functional groups and charges, although without significant textural changes. In addition, organic acids such as acetic and citric acids have been reported to enhance the formation of surface functional groups but at the expense of porosity.^[^ ^32^ ^]^

Biological pretreatment employs biological species, such as bacteria for anaerobic digestion, to produce biochar with specific surface areas. This method is particularly beneficial for feedstocks such as sludge, animal waste, and sugar beet tailings. It also offers a safe disposal option for hyperaccumulator plants that accumulate heavy metals. Other methods involve using metal nanoparticles, carbon nanotubes (CNTs), and graphene as scaffolds to develop carbonaceous material‐supported composites, primarily aimed at enhancing mechanical strength and improving electrical and thermal properties.^[^ ^33^ ^]^ Following the pretreatment, the biomass and its hybrid composites undergo thermal carbonization, converting them into biochar.

### 2.2 Thermal Decomposition Process

#### 2.2.1 Pyrolysis

Pyrolysis is a thermal decomposition process used to convert biomass into biochar and other by‐products. This method traces back to ancient Egypt, where pyrolysis was employed to produce tar or other embalming agents.^[^ ^34^ ^]^ During pyrolysis, dried and ground biomass is subjected to temperatures ranging from 400 °C to 800 °C in an oxygen‐deficient environment. Approximately 50% of the carbon present in the biomass is preserved in the biochar, effectively shielding it from microbial decomposition.^[^ ^35^ ^]^ Biomass pyrolysis is recognized as a “carbon‐negative” pathway because it induces a carbon reversal cycle by converting organic compounds into refractory organic forms, thereby extracting them from the active carbon pool.^[^ ^36^ ^]^ In addition, biomass pyrolysis provides advantages such as enhanced soil organic carbon levels and reduced greenhouse gas emissions.^[^ ^37^ ^]^

There are two types of pyrolysis: slow and fast, each yielding different physicochemical properties in the biochar and by‐products. Slow pyrolysis, also known as conventional pyrolysis, involves heating the biomass at a rate of 1–10 °C min^−1^ to temperatures between 250 °C and 600 °C.^[^ ^38^ ^]^ This approach yields significant amounts of biochar and VOCs; while, generating low concentrations of gases and liquids. The vapors generated during slow pyrolysis have residence times ranging from 5 to 30 min.^[^ ^39^ ^]^ In contrast, fast pyrolysis involves rapid heating rates exceeding 50 °C min^−1^ to temperatures above 600 °C, using dry feedstocks with less than 10% moisture content. This method features a considerably shorter vapor residence time of 1–5 s.^[^ ^40^ ^]^ Fast pyrolysis is typically used to produce higher concentrations of liquid products, such as biofuels, and yields biochar with low VOCs and a higher content of long‐chain hydrocarbons.^[^ ^41^ ^]^

#### 2.2.2 Carbonization

Hydrothermal carbonization converts invasive aquatic plants into porous biochar, which is effective for removing diethyl phthalate from water.^[^ ^42^ ^]^ This process involves heating biomass suspended in water at temperatures ranging from 180 °C to 220 °C under high pressure for extended periods. The exothermic reaction primarily involves dehydration and decarboxylation, leading to a decrease in the oxygen and hydrogen content of the biomass.^[^ ^43^ ^]^

Flash carbonization occurs in a packed biomass bed under pressures ranging from 1 to 2 MPa. The carbonization process begins at the bottom of the bed and progresses upward, with air flowing downward, causing the reactor bed temperature to increase to 300–600 °C. This technique effectively generates biochar with distinct properties suitable for diverse environmental applications.^[^ ^44^ ^]^

#### 2.2.3 Gasification

Gasification is a thermochemical process that converts biomass into gaseous products; while, minimizing CO_2_ emissions. By controlling parameters such as temperature, pressure, gas composition, fuel source, and residence time, the process can be tuned to yield specific products such as solid residues, tar, or gas. Feng et al. investigated gasification using CO_2_ and H_2_O in the presence of alkali and alkaline earth metals on biochar. They found that the reaction predominantly takes place at the solid–gas interface when CO_2_ is utilized, whereas with H_2_O, the reaction is distributed throughout the biochar. This distinction highlights the importance of selecting appropriate gasifying agents to optimize the efficiency and output of the gasification process.^[^ ^45^ ^]^

#### 2.2.4 Torrefaction and Rectification

Torrefaction is a thermochemical process that converts biomass into valuable products such as biochar, biofuel, and biogas. Similar to pyrolysis, it occurs in the absence of oxygen and involves dehydration, carbonization, and caramelization at temperatures ranging from 200 °C to 300 °C. However, torrefaction typically yields carbon material with inferior physicochemical properties compared to that obtained through pyrolysis. There are two types of torrefaction: dry and wet. Dry torrefaction produces a higher volume of biochar but of relatively lower quality, whereas wet torrefaction produces higher‐quality biochar but in smaller quantities. Dry torrefaction is less expensive and does not benefit significantly from catalyst use, whereas wet torrefaction is more effective with catalysts but is costlier and more complex to implement on a mass scale.^[^ ^46^ ^]^ Another variation, steam torrefaction, involves steam explosions at high temperature and pressure. Although this method can enhance the quality of the biochar, it is energy‐intensive and less economically viable for large‐scale operations. These variations in torrefaction processes highlight the trade‐offs among cost, quality, and scalability in biomass conversion technologies.^[^ ^47^ ^]^

### 2.3 Post‐Treatments

The post‐treatment of biochar involves a series of processes applied to biochar after its initial production to enhance or modify its properties for specific applications. These treatments can include physical, chemical, and biological methods that aim to improve biochar's surface area, porosity, functional group presence, adsorption capacity, and overall effectiveness in various environmental applications. Post‐treatment processes may involve techniques such as steam activation, chemical impregnation, biological inoculation, magnetization, and ball milling, among others. The goal of post‐treatment of biochar is to provide significant additional agronomic benefits compared to the use of unprocessed (raw) biochar to meet the requirements of targeted uses, such as pollutant removal, soil amendment, carbon sequestration, and water purification.

#### 2.3.1 Physical Modification

Physical modification of biochar involves processes such as water quenching, leaching, grinding, and sieving to reduce particle size. Pelletization and granulation are also performed to minimize dust and erosion, making biochar easier to handle for specific applications. Further, physical activation methods can significantly alter biochar properties, such as porosity, functional groups, polarity, and hydrophobicity. This method is considered environmentally friendly compared to other modification techniques as it utilizes steam activation and CO_2_‐based pyrolysis of sludge. During combustion, steam and CO_2_ create abundant micropores within the biochar framework. In addition, impurities or organic residues trapped in these pores can be eliminated through steam or gas activation, thereby significantly increasing the specific surface area of biochar. Steam or CO_2_ activation modifies the surface characteristics of biochar, thereby improving its capacity to interact with pollutants such as NO and CH_4_.^[^ ^13^, ^48^ ^]^ Other commonly used oxidizing agents, such as ozone and high‐temperature air, gasify carbon atoms in the biochar structure, thereby opening and expanding the pores.

Additional physical methods for modifying biochar include magnetization, ball milling, and microwave irradiation. Magnetization involves incorporating magnetic oxides, such as iron oxides and cobalt oxides, into the biochar, which significantly enhances its adsorption capacity.^[^ ^49^ ^]^ Ball milling, a simple technique, modifies the microstructure of biochar through the mechanical energy generated from collisions. This process breaks down the biochar into smaller particles, increases its surface area, and introduces new functional groups, including oxygen‐containing groups. Further, biochar can be functionalized during the ball milling process using specific metal reagents or halogen gases, customizing its properties for specific applications.^[^ ^50^ ^]^ Microwave irradiation rapidly heats the biochar to 200–300 °C, resulting in a high surface area and the introduction of hydroxyl groups. When combined with steam activation, microwave treatment further enhances the physicochemical properties of biochar. Through these various physical activation methods, the effectiveness of biochar for environmental applications significantly improves, making it a versatile and valuable material for pollutant removal, soil amendment, and other uses.^[^ ^51^ ^]^

#### 2.3.2 Chemical Modification

Among various methods, chemical post‐treatment or chemical activation is often preferred due to its economic and straightforward approach. This method involves the use of corrosive agents such as acids and alkalis, as well as strong oxidizing agents such as hydrogen peroxide (H_2_O_2_). Chemical post‐treatment enables precise control over the introduction of various functional groups to biochar, thereby enhancing its physicochemical properties, including adsorption capacity, porosity, and overall effectiveness for specific applications. For example, H_2_O_2_ treatment of biochar has been shown to enhance heavy metal sorption through the introduction of oxygen‐containing functional groups, with similar results reported in other studies focusing on toxic metal sorption.^[^ ^52^ ^]^ Beyond increasing surface oxidation, the H_2_O_2_ treatment can also elevate the specific surface area of biochar.^[^ ^53^ ^]^ Similarly, acid treatments using HNO_3_, H_2_SO_4_, and H_3_PO_4_ have demonstrated improvements in the surface properties of biochar, such as lowering pH, increasing oxygen‐functional moieties, removing mobile organic and inorganic mineral components from its matrix, and enhancing the porosity.^[^ ^54^ ^]^ Treatment with bases, such as KOH, sodium hydroxide (NaOH), or potassium carbonate (K_2_CO_3_), also enhances the specific surface area and increases the presence of oxygen‐containing functional groups; although, the treatment conditions significantly influence the outcomes.^[^ ^55^ ^]^ In addition, modifying biochar chemically can enhance its structural stability and resistance to degradation, thereby improving its longevity and effectiveness in long‐term applications.

#### 2.3.3 Biological Modification

Biochar is inherently rich in nitrogen and carbon, with a high surface area and pore volume, making it an ideal medium for microbial growth and reproduction.^[^ ^56^ ^]^ Moreover, biochar serves as an intermediary in facilitating microbial electron transfer because of its redox properties.^[^ ^57^ ^]^ Biological post‐treatment can further enhance the physicochemical properties of biochar by employing microorganisms or enzymes, thereby providing an environmentally friendly alternative to chemical or physical modifications. This method usually consumes less energy and produces fewer hazardous by‐products, thereby aligning with sustainable practices.

Microbial treatments detoxify biochar by breaking down harmful organic compounds, thereby reducing the risk of introducing toxic substances into the environment. In addition, they enhance the surface area and porosity of biochar by decomposing organic residues, thereby increasing its capacity for pollutant adsorption.^[^ ^58^ ^]^ Certain bacterial strains can introduce functional groups onto the biochar surface, enhancing its interaction with and removal of pollutants from water. Enzymatic treatments, on the other hand, facilitate the breakdown of complex organic molecules on the biochar surface, creating more reactive sites for effective pollutant adsorption.^[^ ^59^ ^]^

For water purification, biological post‐treatment is commonly used to address organic pollutants, utilizing microorganisms such as fungi, bacteria, and algae.^[^ ^60^ ^]^ This biological modification involves trapping bacteria within biochar, where they multiply under optimal conditions, including suitable temperature and nutrient availability. A similar process occurs in activated carbons, where microorganisms first biodegrade portions of organic material into biomass, CO_2_, or waste; and then, occupy the adsorption sites. These microorganisms are particularly effective against lipophilic pollutants, enabling them to accumulate and propagate within the biochar structure.^[^ ^61^ ^]^ This approach targets various common pollutants, such as organic compound residues such as dichlorvos, dichlorodiphenyltrichloroethane (DDT), polycyclic aromatic ketones, and polycyclic aromatic hydrocarbons (PAHs), as well as heavy metals such as chromium (Cr), cadmium (Cd), and lead (Pb).^[^ ^62^ ^]^ For example, Du et al. demonstrated that coupling biochar with microorganisms, such as the strain *Sphingomonas sp*. DZ3, significantly enhanced the removal of the pollutant 4‐monobrominated diphenyl ether (BDE‐3).^[^ ^63^ ^]^ A similar study by Qi et al. involved using mixed bacterial cultures (MB9), comprising strains of *Bacillus subtilis, Bacillus cereus*, and *Citrobacter sp*., in combination with biochar derived from pyrolyzed sugarcane bagasse, cattle manure, herb residue, distillers’ grains, and corn stalks. This approach effectively adsorbed heavy metals such as uranium (U) and Cd from the environment.^[^ ^64^ ^]^

## 3 Application of Biochar in Wastewater Pollutant Removal

Biochar plays a crucial role in removing pollutants from wastewater due to its distinct physicochemical properties, which include a high surface area, porous structure, and diverse functional groups. These characteristics render biochar a highly efficient adsorbent for a wide range of contaminants, including organic pollutants (e.g., dyes, pesticides, and pharmaceuticals), inorganic contaminants such as heavy metals (e.g., Cr, Cd, and Pb), and nutrients (e.g., nitrates and phosphates). The tunable porosity and surface functionality of biochar enable customization for specific applications, thereby improving its effectiveness in water pollutant removal. In addition, biochar has demonstrated effectiveness in addressing emerging pollutants, such as microplastics and VOCs.^[^ ^65^ ^]^ This section discusses the application of biochar for the removal of a range of contaminants from wastewater.

### 3.1 Organic Pollutants

#### 3.1.1 Dyes

Dyes or colorants found in industrial wastewater effluents are typically toxic compounds, often acidic or basic, that impart a distinct color to water. These dyes are generally resistant to degradation, remaining stable even when exposed to light, oxidizing agents, or anaerobic conditions, making their removal from water challenging. One commonly studied method for treating dye‐contaminated wastewater is the use of photocatalysts, such as titanium dioxide (TiO_2_). However, this approach has drawbacks, including challenges related to the recovery and reusability of the catalyst.^[^ ^66^ ^]^ Khataee et al. modified biochar using TiO_2_ for sonocatalytic degradation of Reactive Blue 69 (RB69). The modified TiO_2_ demonstrated an increased removal efficiency of up to 98%, creating a good synergy with TiO_2_ that enhanced cavitation bubbles during sonication and allowed for the generation of more hydroxyl radicals. In addition, the presence of functional groups imparts a positive surface charge to biochar, facilitating the adsorption of anionic dyes. The mechanism involves interaction of •OH, O_2_^−^, h^+^, and OH^−^ with h^+^ and free •OH radicals.^[^ ^67^ ^]^

Other widely studied techniques for dye removal include coagulation, advanced oxidation processes (AOPs), oxidation, membrane processes, nanofiltration, forward osmosis, and biological methods utilizing microorganisms such as bacteria.^[^ ^68^ ^]^ Among these, adsorption emerges as an efficient method enabling the treatment of large volumes of water without producing harmful residues or by‐products. Recently, biochar as a bio‐adsorbent has gained attention as a green alternative and is recognized as an environmentally friendly and cost‐effective option for dye adsorption. The high surface area, adsorption capacity, low cost, and availability of biochar render it particularly attractive for this application. Several factors influence the efficacy of dye adsorption on biochar.

##### Biochar Characteristics

The properties of biochar are largely determined by the feedstock from which it is derived and the conditions during the conversion process. For example, biochar produced at higher pyrolysis temperatures typically exhibits lower H/C and O/C ratios, higher surface area, hydrophobicity, and microporosity. The presence of minerals and functional groups in biochar enhances activities such as precipitation and cation exchange.^[^ ^69^ ^]^

##### Solution pH

The pH of the solution is a critical factor that directly correlates with the adsorption capacity of biochar. The surface chemistry of biochar, influenced by pH, determines the availability of hydrogen ions and adsorbate ions for binding at the active sites on the biochar surface.^[^ ^70^ ^]^ For example, Babaei et al. observed that the adsorption of methylene blue using biochar increased from 40% to 90% as the pH varied from 2 to 9. This behavior was attributed to the negatively charged surfaces of biochar that promote electrostatic interactions.^[^ ^71^ ^]^

##### Biochar Dosage

Increasing the biochar concentration initially enhances the adsorption rate by increasing sorption site availability. However, this trend diminishes as the reaction proceeds. A lower amount of biochar leads to greater removal efficiency per unit weight, possibly facilitating dye molecule access to sorption sites.^[^ ^72^ ^]^

##### Temperature

The adsorption rate is directly influenced by the temperature, up to an optimal point. Satish Kumar et al. conducted adsorption experiments at temperatures ranging from 20 °C to 55 °C and found that maximum dye removal occurred at temperatures between 35 °C and 40 °C.^[^ ^73^ ^]^

##### Dye Concentration and Contact Time

Initially, increasing the dye concentration improves adsorption by increasing the frequency of collisions between dye anions and the biochar surface. However, at higher concentrations, the adsorption capacity decreases due to the saturation of sorption sites on the biochar. Consequently, lower dye concentrations result in a higher removal rate because the ratio of dye molecules to sorption sites is reduced.^[^ ^74^ ^]^

#### 3.1.2 Organochlorine Compounds

Chlorinated persistent organic pollutants are organic compounds in which a hydrogen atom in a carbon or hydrocarbon structure is substituted by a chlorine atom. Examples include organochlorine pesticides (OCPs), trichloroethene (TCE), polychlorinated biphenyls (PCBs), tetrachloroethene (PCE), triclosan (TCS), and chlorobenzenes (CBs).^[^ ^75^ ^]^ The removal of these pollutants from wastewater usually necessitates biochar specifically modified to target the particular characteristics of these chlorinated compounds.

Trichloroethylene (TCE), an industrial solvent and a chlorinated aliphatic hydrocarbon, contaminates water and air, thereby entering the human body. TCE is carcinogenic and toxic to human organs, including during pregnancy. The effective removal of TCE depends on the biochar characteristics, such as small pore size, a high number of C─O functional groups, and a large specific surface area.^[^ ^76^ ^]^ Studies have shown that smaller biochar particle sizes, increased microporosity, and enhanced electrical conductivity enhance TCE removal.^[^ ^77^ ^]^ Chemically modified biochar, such as biochar combined with nanoscale zero‐valent iron (nZVI), can enhance TCE degradation through functional groups that act as electron mediators and produce reactive radicals such as sulfate and hydroxyl radicals.^[^ ^78^ ^]^ In addition, coupling biochar with biological agents promotes anaerobic TCE degradation by facilitating microbial electron transfer, reducing toxic inhibition, and establishing a dichlorination system by encouraging the growth of co‐metabolizing microorganisms.^[^ ^79^ ^]^

Polychlorinated biphenyls (PCBs) have been extensively utilized in industrial products, such as lubricants, rubber, fireproofing agents, and printing inks, due to their chemical and physical stability, as well as their resistance to acids and bases. However, PCBs are challenging to naturally degrade and have contaminated water, soil, and sediments, posing risks to human health and the environment. The adsorption of PCBs by biochar depends on the preparation method, impacting crucial properties such as specific surface area, pore volume, functional groups, hydrophilicity, and exchange properties. For example, Silvani et al. demonstrated that the effectiveness of PCB adsorption is more dependent on the type of biochar used rather than its quantity.^[^ ^80^ ^]^ Li et al. further enhanced biochar by modifying it with laccase immobilization for PCB treatment, thereby successfully creating a reusable and green adsorbent capable of enzymatically degrading 4‐hydroxy‐3,5‐dichlorobiphenyl (HO‐DiCB) in wastewater.^[^ ^81^ ^]^

Triclosan (TCS, 5‐chloro‐2‐[20,40‐dichlorophenoxy]), a chlorinated phenoxyphenol, has been widely used in personal care products (PPCPs) such as cosmetics, soaps, and household items since 1957. TCS has been detected in water, soil, and humans, and is recognized for its ability to disrupt endocrine and reproductive systems in mammals. Owing to its high internal surface tension and low solubility, TCS is highly stable and resistant to natural degradation. Researchers have investigated various biochar modifications to enhance the adsorption of TCS from wastewater. For example, biochar derived from food waste and pyrolyzed at 300 °C demonstrated a high adsorption capacity due to its large surface area and the presence of diverse functional groups, such as aldehydes.^[^ ^82^ ^]^ Acidic modification with HCl further enhances the biochar by introducing functional groups, including carboxylic (C─O) bonds and phenolic C─H groups, onto its surface. These modifications facilitate the binding of TCS phenyl groups to the phenol groups on the biochar through hydrogen bonding and π─π stacking interactions, thereby significantly increasing its adsorption capacity.^[^ ^83^ ^]^ Another study demonstrated that activated sodium persulfate can produce reactive species, such as radicals and singlet oxygen, which attach to the biochar surface and facilitate electron transfer. This process results in the formation of carbon‐centered radicals that contribute to the mineralization of TCS.^[^ ^84^ ^]^ Additional methods employed to remove TCS from wastewater include coupling biochar with photocatalysts to form composites, such as biochar/Ag_3_PO_4_/polyaniline, and modifying biochar through nitrogen and BiVO_4_ doping.^[^ ^85^ ^]^

Chlorobenzene (CB), an industrial solvent commonly used as an insect repellent and an intermediate in pesticide production, is highly toxic and volatile. Its ease of migration and potential for biomagnification pose significant risks to both the environment and human health. Shang et al. prepared a hybrid composite of biochar with nanoscale zero‐valent iron (nZVI) and palladium (Pd) to remove 1,2,4‐trichlorobenzene (1,2,4‐TCB) from contaminated environments. To enhance the physicochemical properties of the biochar, including its aromaticity, specific surface area, and hydrophobicity, the biochar was treated with hydrofluoric acid (HF) and sodium hydroxide (NaOH). This treatment improved the interaction between the modified biochar and CB; while, also increasing the number of sites available for iron (Fe) and palladium (Pd) nanoparticle support within the composite.^[^ ^86^ ^]^

#### 3.1.3 Phenols

Phenol, an aromatic compound and a major constituent of organic wastewater, is highly carcinogenic and poses significant health risks even at low concentrations, potentially causing severe damage to the eyes, skin, heart, respiratory system, and nervous system. Owing to the stability and persistence of its aromatic ring structure, phenol is highly mobile in the environment and can easily accumulate in the food chain. Various strategies such as reverse osmosis, ion exchange, and extraction have been employed to remove phenols from wastewater, but few have proven highly effective. Among these, AOPs are particularly effective in targeting a wide range of toxins by oxidizing them into less harmful compounds. In this context, biochar plays a versatile role—not only serving as a catalyst or catalyst support in AOPs but also activating oxidants such as hydrogen peroxide (H_2_O_2_) and persulfates, which are commonly used in these processes. In addition, biochar helps stabilize the reactive species generated during AOPs, preventing their premature decomposition and ensuring their availability for pollutant degradation. It also reduces harmful by‐products, improving the treatment process's overall efficiency and safety.

**Figure 3:**
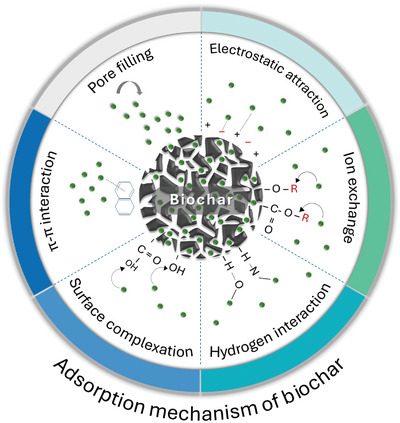
Mechanisms responsible for contaminant adsorption on biochar.

Xie et al. investigated the use of biochar derived from cherry stones, nitrogen‐doped by mixing it with melamine, and activated it with KOH. This nitrogen‐doped biochar was then employed as an activator for peroxymonosulfate to degrade phenols.^[^ ^87^ ^]^ Similarly, Lingamdinne et al. performed KOH activation on biochar derived from sunflower stems, achieving a surface area of 452 m^2^ g^−1^, with phenol and bisphenol A (BPA) uptake capacities of 333 and 366 mg g^−1^, respectively. The improved adsorption was attributed to synergistic effects, including hydrogen bonding, electrostatic attraction, and π–π stacking between the adsorbate and the adsorbent.^[^ ^88^ ^]^ In addition to KOH activation, H_2_O_2_ had been utilized as an activator for sludge‐derived biochar, which was loaded with bimetallic Cu and Ni to form a composite. Liu et al. reported a 100% removal and 69% mineralization of phenol using this biochar in a Fenton‐like process, facilitated by efficient electron transfer through the valence state conversion cycles among Cu(0)–Cu(II), Ni(III)–Ni(II), and Cu–Ni.^[^ ^89^ ^]^ Shan et al. developed a Fe_3_O_4_‐modified magnetic porous loofah biochar that effectively removed phenol through hydrogen bonding, π–π conjugation, and pore‐filling mechanisms. This approach enabled monolayer adsorption and chemisorption, achieving an adsorption capacity of 39.4 mg g^−1^ and maintaining up to 78% efficiency after seven cycles.^[^ ^90^ ^]^ The various mechanisms involved in these processes are detailed in **Figure** **3**.

The mechanisms following phenol adsorption can be attributed to hydrogen bonding, electrostatic interactions, and π–π interaction. Under neutral conditions, a positively charged biochar acts as a π‐electron donor to attract phenols with electron‐rich benzene rings acting as π‐electron acceptors, creating an electrostatic interaction that absorbs phenols. Moreover, H‐bonding is another strong interaction mechanism between phenolic groups and biochar. This occurs through two pathways: i) interaction between the hydroxyl groups on the biochar surface acting as H‐donors and the O or N in the phenols as H‐acceptors and (ii) interaction between the aromatic rings in the phenol group and the hydroxyl group.^[^ ^8b^ ^]^

#### 3.1.4 Polycyclic Aromatic Hydrocarbons

PAHs are compounds comprising two or more aromatic rings, typically present in crude oils and petroleum products. They are generated during the combustion of organic matter at temperatures above 700 °C. PAHs can be transported through air and water, resulting in their occurrence in wastewater effluents, industrial discharges, and areas associated with fossil fuel production and leakages.^[^ ^91^ ^]^ These compounds are known for their carcinogenic and mutagenic properties, presenting substantial hazards to the environment and human health.

Traditional chemical treatments for PAHs often involve hazardous reagents, leading to potential secondary pollution. Physical methods, such as using magnetic sorbents and carbon nanotubes, have been explored; however, these approaches suffer from limitations including extended extraction times, low recovery rates, and economic impracticality for large‐scale applications.^[^ ^92^ ^]^ In contrast, biochar has emerged as an effective adsorbent for PAHs. For example, Jesus et al. utilized biochar derived from coconut and orange waste to adsorb PAHs, leveraging hydrophobic and π–π interactions between the adsorbate and the adsorbent. The removal efficiency varied from 23% to 84%, depending on the characteristics of the biochar.^[^ ^91^ ^]^ Rashad et al. treated a mixture of PAHs, including naphthalene, phenanthrene, and anthracene, using various forms of biochar, both activated and non‐activated. They observed that acid‐treated biochars exhibited the best performance, attributing this to the increased surface area post‐activation.^[^ ^92^ ^]^ Qiao et al. investigated biochar derived from the algae *Enteromorpha prolifera* for PAH degradation, specifically targeting pyrene (PYR) and benzo[a]pyrene (BaP) and achieving a degradation efficiency of ≈87–90%. Several factors influence PAH adsorption on biochar, including pH, which affects interactions such as hydrophobicity, π–π interactions, and donor–acceptor interactions. An optimal pH range of 6–8 has been reported for achieving the most efficient biodegradation. In addition, critical factors include biochar dosage, initial PAH concentration, and contact time.^[^ ^93^ ^]^

### 3.2 Inorganic Pollutants

#### 3.2.1 Nitrate

Inorganic nitrogen, primarily in the form of nitrates (NO_3_^−^), is commonly present in soil and is widely used as a fertilizer in agriculture. Nitrates, due to their water solubility, can easily leach from the soil into groundwater, resulting in elevated concentrations that pose risks to ecological balance and public health. The World Health Organization (WHO) has set the maximum allowable limit for nitrate in drinking water at 50 mg L^−1^.^[^ ^94^ ^]^ Adsorption is the preferred method for nitrate removal due to its simplicity, effectiveness, repeatability, and economic viability. Biochar, a carbon‐rich material derived from inexpensive biomass sources, has gained attention as an effective adsorbent for this purpose. For example, Wang et al. developed biochar from apple branches and enhanced its adsorption capacity by physically activating it with Mg/Al layered double hydroxide. They observed a significant improvement in nitrate adsorption, from 17% to 83%, after activation. The adsorption process was attributed to multiple physicochemical mechanisms, including physical adsorption, intraparticle diffusion, ion exchange, metal‐bonded bridges, and electrostatic interactions.^[^ ^95^ ^]^ Guo et al. reported the use of biochar‐amended constructed wetlands for nitrate removal, where they identified the microbial metabolic pathways responsible for nitrate reduction.^[^ ^96^ ^]^ The study demonstrated that biochar addition enhanced nitrate removal by increasing the activities of dehydrogenase (DHA), denitrifying enzymes, and enhancing the electron transport system. Moreover, biochar promoted the availability of genes encoding enzymes involved in ATP production and electron generation, transportation, and consumption, thereby improving the overall efficiency of the constructed wetland. In addition, Zhang et al. utilized biochar immobilized with bacterial cells of *Pseudomonas mendocina* GL6 to enhance nitrate removal from wastewater.^[^ ^97^ ^]^ This enhancement was associated with the increased expression of denitrification functional genes (*napA* and *nirK*) and electron transfer genes (*napB* and *napC*) involved in the denitrification process, leading to more effective nitrate removal from the water.

#### 3.2.2 Ammonia

Ammonium compounds are inorganic, non‐volatile substances that are highly soluble in water. Even low concentrations of ammonia, as little as 0.2 mg L^−1^, can be toxic to living organisms, contributing to eutrophication and inhibiting critical processes such as photosynthesis in algae and protein metabolism in aquatic animals. Ammonia is primarily released into the environment through agricultural and livestock effluents, industrial sewage, and the production of fertilizers, textiles, and refined oil. It is also widely used in agriculture as a component in fungicides, herbicides, pesticides, and microbiocides.^[^ ^98^ ^]^

Various physical, chemical, and biological methods, such as membrane separation and precipitation, have been developed to remove ammonium from water. However, adsorption stands out for its simplicity, cost‐effectiveness, and environmental friendliness. Although materials such as zeolites and molecular sieves have been explored for this purpose, biochar, a stable carbon‐rich material derived from organic wastes, presents a promising alternative.

A study on biochar derived from almond kernels, activated with KOH and dolomite, reported maximum adsorption uptakes of 36.3 and 40.6 mg g^−1^, respectively.^[^ ^98^ ^]^ Similarly, Feng et al. investigated biochar sourced from coconut shells, rice straw, and wood to treat anaerobic digestion slurry with high ammonium content.^[^ ^99^ ^]^ Their study identified key parameters in the adsorption process, such as H/C (aromaticity), O/C (hydrophilicity), pH, electrical conductivity, and ash content. They concluded that physical adsorption was the primary mechanism, with additional contributions from ion exchange and the presence of functional groups that enhanced biochar's ability to adsorb ammonium.

#### 3.2.3 Phosphates

Phosphorus is commonly found in wastewater discharges from agriculture, chemical treatment plants, and households, particularly due to the use of detergents. While phosphorus is essential for plant growth in appropriate amounts, excessive concentrations can cause eutrophication in water bodies.^[^ ^100^ ^]^ Moreover, phosphorus is a finite resource, emphasizing the importance of its recovery for sustainable nutrient management. Conventional phosphorus removal methods, such as crystallization, precipitation, and enhanced bioprocess treatments, are often chemical‐intensive, expensive, and time‐consuming. In contrast, adsorption provides a cost‐effective and straightforward alternative. Common adsorbent materials include biological substances such as bacteria and minerals such as alum and iron.^[^ ^101^ ^]^

Biochar, with its high surface area, pore structure, surface functional groups, and ion exchange capacity, is an ideal candidate for phosphorus removal from water. For example, Park et al. investigated phosphate adsorption using biochar derived from crawfish shells and found that adsorption capacity increased with higher pyrolysis temperatures.^[^ ^100^ ^]^ They concluded that the adsorption was strongly influenced by the calcium content in the crawfish biochar and the pH of the solution. Specifically, crawfish biochar produced at 600 °C exhibited higher adsorption in acidic or neutral pH conditions; while, that produced at 800 °C was more effective in alkaline environments. The adsorption mechanisms involved calcium from the biochar surface interacting with phosphate hydrolysis products such as H_2_PO_4_^−^ and HPO_4_^2−^ or precipitation processes between PO_4_^3−^ and dissolved calcium from CaO and Ca(OH)_2_. Li et al. further explored biochar derived from egg and oyster shells, which contain varying concentrations of calcium.^[^ ^102^ ^]^ Their study demonstrated that phosphate adsorption occurred through a ligand exchange process, where phosphate ions replaced carbonate ions, forming Ca–PO_4_ complexes. The pH of the solution significantly influenced the adsorption process as different phosphate species (H_2_PO_4_^−^, HPO_4_^2−^, and PO_4_^3−^) dominate at different pH levels. For example, H_2_PO_4_^−^ is predominant at a pH range of 2.13–7.2, HPO_4_^2−^ from 7.2 to 12.3, and PO_4_^3−^ beyond pH 12.3. The affinity of calcium for phosphate ions follows the order PO_4_^3−^ > HPO_4_^2−^ > H_2_PO_4_^−^, indicating that higher pH levels are more favorable for adsorption. The maximum adsorption capacities reported for the egg and oyster shell‐based biochars were 154 and 129 mg g^−1^, respectively.^[^ ^102^ ^]^

#### 3.2.4 Fluoride

Fluoride naturally occurs in the earth's crust in the form of minerals such as fluorite, topaz, biotite, fluorspar (CaF_2_), fluorapatite (Ca_10_F_2_(PO_4_)_6_), and cryolite (Na_3_AlF_6_). Natural processes such as weathering and dissolution, along with human activities, release fluoride ions into the environment. Globally, chronic exposure to high fluoride levels poses significant risks to human health and the environment, with industrial discharges from sectors such as semiconductors, fertilizers, glass, and steel contributing to contamination. The WHO recommends a permissible limit for fluoride in water ranging from 0.5 to 1.5 mg L^−1^. While low doses of fluoride are beneficial—commonly added to pharmaceutical products and dental care items to prevent tooth decay and osteoporosis—excessive exposure can lead to adverse health effects, including joint deformation, skeletal and dental fluorosis, and neurotoxicity in children.^[^ ^103^ ^]^ In this context, biochar emerges as a promising alternative, serving as both a catalyst support and an effective adsorbent material.

Li et al. demonstrated the utilization of sewage sludge‐based biochar, amended with aluminum and yttrium (Al/Y), for fluoride removal from wastewater over a wide pH range, achieving a maximum adsorption capacity of 62.4 mg g^−1^.^[^ ^104^ ^]^ The incorporation of Y and Al improved the adsorption process by enhancing the affinity for fluoride ions; while, the intrinsic dispersing effect of biochar facilitated better access to active sites, thereby enhancing defluoridation. Similarly, Khan et al. employed pinecone‐derived biochar modified with Fe and Al salts under varied conditions, including pH, contact time, adsorbent dose, and initial fluoride concentration. The biochar modified with AlCl_3_ demonstrated a maximum adsorption capacity of 14.1 mg g^−1^ in spiked water and 13.1 mg g^−1^ in in situ groundwater.^[^ ^105^ ^]^ A study on fluoride removal using red algae‐derived biochar modified with Fe_3_O_4_ nanoparticles was conducted. This modified bio‐adsorbent exhibited a maximum adsorption capacity of 96.4 mg g^−1^ and could be rapidly separated under an external magnetic field.^[^ ^106^ ^]^ Sadhu et al. also reported defluoridation using biochar derived from watermelon rind (*Citrullus lanatus*), with a maximum capacity of 9.5 mg g^−1^.^[^ ^107^ ^]^

The fluoride adsorption mechanism is mainly influenced by the biochar material. For example, plant‐based biochar primarily relies on electrostatic adsorption and ion‐exchange processes, whereas animal waste‐based biochar follows a co‐precipitation mechanism. Modifications, such as incorporating metal ions, enhance electrostatic adsorption and ion exchange. In addition, doping biochar with biomaterials introduces new mechanisms, such as ion‐pair complexation and site replacement.^[^ ^108^ ^]^

#### 3.2.5 Heavy Metal Removal

Common heavy metal pollutants, such as lead (Pb), chromium (Cr), cadmium (Cd), and arsenic (As), are non‐biodegradable and naturally sourced from the earth's crust. These metals can contaminate water through natural processes such as weather and volcanic eruptions, as well as through anthropogenic activities, including agricultural runoff, industrial processes such as metal refining, electronics manufacturing, coal power generation, and petroleum combustion. Heavy metals are highly toxic, and chronic exposure can result in serious health hazards, including carcinogenic effects.^[^ ^109^ ^]^

Biochar presents a more viable option for removing heavy metals via adsorption due to its cost‐effectiveness, renewability, environmental sustainability, and high efficiency. The adsorption mechanism depends on several factors such as pore structures, surface functional groups, solution pH, and the interaction between the adsorbent (biochar) and the adsorbate (type of pollutant). These mechanisms can include physical and chemical adsorption, ion exchange, electrostatic interactions, precipitation, and complexation. The high specific surface area and network of meso‐, micro‐, and macropores in biochar enhance its affinity for metal adsorption. For example, electrostatic interactions between the negatively charged biochar surface and positively charged metal ions, as well as ligand formation, contribute to effective metal adsorption.^[^ ^110^ ^]^

Thaci et al. utilized biochar derived from pine cones to remove Pb(II) and Cd(II) ions, achieving maximum adsorption capacities of 43.5 and 32.9 mg g^−1^ at pH 6 and 7, respectively.^[^ ^111^ ^]^ They noted that adsorption occurred more rapidly for single‐ion removal than for co‐existing ions, and regeneration was possible using 0.5 m HNO_3_. Similarly, Das et al. employed biochar from black gram, pine needles, Lantana camara, and maize stalk to extract heavy metals such as Cd, Pb, Ni, Zn, Cu, and As, with removal rates ranging from 45.5% to 66.1%.^[^ ^112^ ^]^ Their study revealed that adsorption efficiency decreased with the increasing metal concentration due to a decrease in the available active binding sites. Roy et al. generated biochar from in vitro grown shoots and roots of *Plumbago zeylanica* and successfully removed Cd and Cr, achieving a maximum adsorption capacity of 100 ppm.^[^ ^113^ ^]^

To further enhance biochar's capacity for removing heavy metals, modifications can be made. For example, Zhao et al. synthesized nano‐Fe_2_O_3_‐loaded KMnO_4_‐modified loofah biochar for Cu(II) removal.^[^ ^114^ ^]^ Their findings revealed that MnO*x* increased the adsorption capacity; while, KMnO_4_ oxidation enhanced the presence of oxygen‐containing functional groups, resulting in stronger electrostatic attraction, ion exchange, and complexation with Cu(II) ions. Xu et al. found that the sorption of heavy metals such as Cu, Zn, and Cd by biochar derived from dairy manure was primarily due to precipitation with carbonates and phosphates. However, they also observed that 2–25% of metal removal could be attributed to adsorption through complexation with phenolic‐OH sites or delocalized π electrons.^[^ ^115^ ^]^

Using biochar to remove heavy metals from aqueous solution may follow different mechanisms, such as ion exchange, complex formation, and electrostatic interaction. The nature and mechanism of adsorption differ based on biochar type and modifications, such as feedstock and pyrolysis temperature. Ye et al. studied heavy metal removal from wastewater using biochar. They utilized cattle manure and cherry wood as feedstock for preparing the biochar and studied the removal of heavy metals such as Pb, Cd, and Ni. They explored metal ion exchange (*Q*_e_), mineral precipitation (*Q*_p_), functional group complexation (*Q*_f_), and heavy metal–π coordination (*Q*_π_) to determine the reaction mechanism.^[^ ^116^ ^]^ Cattle manure‐based biochar exhibited a *Q*_e_/*Q*_t_ ratio exceeding 40% for Cd and Ni, indicating an ion‐exchange mechanism. For Pb, the *Q*_e+p_/*Q*_t_ ratio reached 79%, indicating mineral precipitation as the dominant mechanism. In contrast, cherry wood‐based biochar showed a decreased *Q*_e+p_/*Q*_t_ ratio of 60%, possibly due to the presence of lower mineral content and higher levels of oxygen‐containing functional groups. Understanding the adsorption mechanism is crucial for optimizing biochar performance. Various pathways have been reported for Cr(VI) removal, including electrostatic attraction, direct reduction, and complexation. Rajapaksha et al. observed direct reduction of Cr(VI) to Cr(III), with an increased observed peak of Cr(III) and reduced Cr(VI) intensity post‐adsorption, and it was analyzed using X‐ray absorption near edge structure.^[^ ^117^ ^]^ On the other hand, Zhou et al. observed electrostatic interaction between Cr(VI) ions and biochar‐containing functional groups, positively charged due to the presence of hydroxyl and carboxyl groups.^[^ ^118^ ^]^

### 3.3 Pharmaceutical Removal

Pharmaceuticals comprise a wide range of biological and non‐biodegradable compounds utilized for treating infections and diseases in humans and animals. These include analgesics, antibiotics, anti‐inflammatory agents, sedatives, birth control hormones, and other pharmacological and personal care products (PPCPs). Designed with specific structures and compositions for targeted absorption and distribution within the body, pharmaceutical contaminants infiltrate water systems through hospital effluents, pharmaceutical industry discharges, agricultural runoff, and household waste, primarily through urine and excreta. Within water bodies, these contaminants can persist as parent compounds, metabolites, or conjugates of glucuronic and sulfuric acid.

The adsorption process is influenced by a combination of mechanisms, such as pore filling, complexation, ion exchange, hydrogen bonding, electrostatic attraction, and π–π interactions (Figure 3). Physical interactions typically involve less polar compounds binding to non‐polar adsorbents, with effectiveness depending on the surface properties of the adsorbent. Both carbonized and non‐carbonized fractions of biochar contribute to these surface properties. The dominant mechanisms in biochar adsorption include π–π interactions and electrostatic attraction, where the aromatic rings in the biochar interact with the aromatic rings in pharmaceutical contaminants, making π–π interactions essential for pollutant removal. In addition, *n*–π interactions occur between electron‐deficient aromatic pharmaceutical compounds and oxygen‐containing functional groups (─COOH and ─OH) on the biochars, which act as electron donors.^[^ ^119^ ^]^

For certain pharmaceuticals such as paracetamol, hydrogen bonding serves as the primary adsorption mechanism. The oxygen and nitrogen atoms in the pharmaceutical compounds and the hydroxyl groups in the biochar act as H‐acceptors and H‐donors, respectively, forming hydrogen bonds through dipole–dipole interactions. Further, the interaction between the aromatic ring and the hydroxyl group in the biochar can result in Yoshida hydrogen bonding.^[^ ^120^ ^]^

The pH of the solution significantly influences the extent of adsorption, particularly in cases involving electrostatic interactions where ionizable functional groups in the adsorbent interact electrostatically with the adsorbate. At pH levels above the pKa of the pharmaceutical compound, the compound becomes ionically negative; while, at pH levels below the pKa, it remains neutral. Increased ionic strength can reduce electrostatic repulsion, thereby decreasing adsorption capacity.^[^ ^121^ ^]^ Another observed mechanism is hydrophobic interaction, where non‐polar groups cluster together when in contact with water. For example, in the case of diclofenac and levofloxacin, Maged et al. found that higher hydrophobicity increased adsorption.^[^ ^122^ ^]^ A study by Guo et al. on tetracycline adsorption over nitrogen‐enriched biochar identified primary adsorption processes as pore filling, Lewis acid–base interactions, and π–π interactions, with some contribution from hydrogen bonding. The presence of graphitic and pyridinic nitrogen content was also found to enhance adsorption.^[^ ^123^ ^]^

To enhance the removal of pharmaceuticals, Fan et al. developed a KOH‐activated biochar composite integrated with MnAl‐layered double hydroxides (LDHs) for tetracycline adsorption.^[^ ^124^ ^]^ This hybrid composite is particularly effective in pharmaceutical removal due to its synergistic properties: the biochar offers a large surface area and porosity, facilitating physical adsorption of contaminants; while, the MnAl‐LDHs introduce additional functional groups and crystalline minerals such as Mn─O and Mn─O─Mn that promote chemical interactions. The biochar composite demonstrated a maximum tetracycline adsorption capacity of 1477.7 mg g^−1^, with enhanced adsorption observed over a pH range between 3 and 9. Similarly, Zou et al. observed analogous adsorption mechanisms in biochar derived from apple branches, which were magnetized and coated with humic acid for norfloxacin removal.^[^ ^125^ ^]^ Peiris et al. investigated the mechanism of tetracycline removal, which involves hydrogen bonding, surface complexation, electrostatic attraction, and cation bridging.^[^ ^126^ ^]^ Tetracycline possesses functionalities that can perform various coupling reactions, including Lewis acid–base and π–π electron donor–acceptor (EDA) interactions with the donating arene rings on the biochar surface. In addition, tetracycline contains moieties such as phenol, enone, amine, and hydroxyl groups that can form H‐bonds with the carboxyl and hydroxyl groups in biochar. Another favorable mechanism for tetracycline adsorption on biochar could be cation exchange, where a positively charged organic molecule displaces naturally occurring cations associated with a negatively charged site on the biochar surface. This phenomenon is commonly observed with molecules having exchangeable cation‐rich surfaces, such as montmorillonite and iron/aluminum hydroxides.^[^ ^127^ ^]^ Other studies have reported the removal of pharmaceutical compounds, including oxytetracycline, diclofenac, ciprofloxacin, levofloxacin, ibuprofen, and amoxicillin, using biochar derived from fish scales, pine needles, rice husks, date palms, and coffee grounds.^[^ ^128^ ^]^

### 3.4 Microplastics and Nanoplastics

With the advent of the industrial revolution, urbanization, and a rapidly growing population, the production of plastics has significantly increased due to their versatility, durability, and stability. However, the non‐biodegradable and persistent nature of plastics has resulted in substantial accumulation of plastic debris, posing a global threat to humans, plants, animals, and the environment. Microplastics, defined as particles smaller than 5 nm and degraded from larger plastics, were first identified by Thompson et al.^[^ ^129^ ^]^ Recently, microplastics have been further categorized into nanoplastics (<1 µm), small microplastics (1–20 µm), and large microplastics (20 µm to 1 mm). These microplastics are ingested by aquatic organisms, entering the human body through the food chain, and have been associated with organ damage and various diseases. Further, microplastics in water can serve as vectors, promoting the spread of pollutants such as organic contaminants, heavy metals, and pharmaceuticals.^[^ ^130^ ^]^

Biochar can be modified to enhance its properties for microplastic removal, and its effectiveness in absorbing microplastics depends on both the characteristics of the biochar and external factors such as pH and temperature. For example, Ganie et al. produced biochar from sugarcane bagasse at various pyrolysis temperatures (350 °C, 550 °C, and 750 °C) to remove nanoplastics.^[^ ^131^ ^]^ The biochar produced at 750 °C exhibited the highest efficiency, achieving over 99% removal within 5 min and an adsorption capacity of 44.9 mg g^−1^. The enhanced adsorption capacity was attributed to the high surface area of 540 m^2^ g^−1^ and the improved porosity of the biochar. However, the presence of humic acid decreased nanoplastic adsorption because of steric hindrance and electrostatic repulsion, resulting from the surface stabilization of nanoplastics and biochar. The adsorption efficiency also varied based on the wastewater source, with a 25% reduction in efficiency attributed to the presence of other organic contaminants and competing ions.

Another effective method for microplastic removal involves the use of magnetized biochar, where magnetic nanoparticles are adsorbed onto the biochar, making the process independent of particle size. While previous methods, such as using magnetic CNTs and Fe‐modified fly ash, have been explored, they often suffer from agglomeration and instability. In contrast, biochar loaded with magnetic substances offers greater stability. For example, Wu et al. prepared biochar from rice husks, an agricultural waste, and loaded it with magnetic substances through an impregnation–pyrolysis process.^[^ ^132^ ^]^ This biochar demonstrated a polystyrene adsorption efficacy of 99.96%. The primary adsorption mechanisms identified were a combination of electrostatic attraction, hydrogen bonding, and π–π interactions, with the most favorable results observed under acidic conditions. Similarly, Singh et al. designed an iron‐modified biochar pyrolyzed at 550 °C and 850 °C that could magnetically extract nanoplastics of different sizes with surface functionalities.^[^ ^133^ ^]^ Some of the dominant mechanisms included surface complexation and electrostatic attraction. They observed that there was little significance of ionic strength and solution pH on nanoplastics removal. After the interaction with nanoplastics, there was an observed change in zeta potential, indicating the electrostatic attraction mechanism at play. In addition, spectroscopic characterization exhibited disappearance of COO^−^ stretching vibrations and conversion of octahedral stretching of the Fe─O magnetite peak and octahedral–tetrahedral Fe─O stretching to γ‐FeOOH after interaction with nanoplastics, which could be due to complexation with the hydroxyl and carboxyl groups in the biochar.

Wang et al. investigated the utilization of corn straw and hardwood biochar as filters to remove microplastic spheres ≈10 µm in size.^[^ ^134^ ^]^ They proposed a mechanism involving being “stuck,” “trapped,” and “entangled,” where the honeycomb and thin chip structures of the biochar improved its removal efficiency, making it suitable for use in sand filters. Using an environmental scanning electron microscope, the researchers immobilized the microplastics by retaining their morphological structure, which retained the microparticles on surfaces, pores, and underneath biochar chips. The study revealed that the “trapped” and “entangled” mechanisms of immobilization offer superior efficiency compared to sand filters of similar size.

Despite its potential, biochar still faces challenges such as recovery and regeneration, secondary pollution from modification strategies, and the proper disposal of adsorbed microplastics. Future modifications, such as impregnating biochar with metals or using it as a carrier for microorganisms, could enhance its stability and make it more suitable for practical applications.

### 3.5 Volatile Organic Compounds

VOCs, both naturally occurring and anthropogenic compounds, are known for their low boiling points, which allow them to exist as gases at normal temperatures and pressures. These compounds include sulfur compounds, amines, phenols, indoles, hydrogen sulfide, volatile fatty acids (VFAs), and inorganic ammonia.^[^ ^135^ ^]^ VOCs are highly toxic, carcinogenic, and present significant environmental risks, including the formation of photochemical smog and contribution to the greenhouse effect. Exposure to VOCs can damage the nervous, gastrointestinal, and endocrine systems, and disrupt various metabolic functions. The Gothenburg Protocol was established to limit VOC emissions due to their propensity to undergo complex photochemical reactions with atmospheric nitrogen oxides, resulting in secondary pollution.^[^ ^136^ ^]^

VOCs can be removed through purification processes or by converting them into non‐toxic compounds. Purification methods include condensation, adsorption, absorption, and membrane separation, whereas conversion methods involve catalytic and thermal combustion, photocatalytic oxidation, and low‐temperature plasma.^[^ ^137^ ^]^ Among these, adsorption is often preferred due to its effectiveness and recyclability. Carbon‐based materials have attracted interest for VOC removal because of their cost‐effectiveness and excellent adsorption properties, with biochar emerging as a particularly effective option.

The adsorption capacity of biochar is highly dependent on its properties, such as surface area, pore volume and structure, and the functional groups attached to its surface. These properties are influenced by the source material of the biochar and the conditions under which it is prepared, such as the pyrolysis temperature. For example, Jayawardhana et al. prepared biochar from municipal waste to remove VOCs such as toluene and m‐xylene, achieving adsorption capacities of 850 and 550 µg g^−1^, respectively, through non‐monolayer adsorption.^[^ ^138^ ^]^ Zhang et al. investigated biochar produced from five different sources, including bamboo, sugarcane bagasse, Brazilian pepper wood, sugar beet tailings, and hickory wood, at pyrolysis temperatures of 350 °C, 450 °C, and 600 °C.^[^ ^136^ ^]^ They found that adsorption capacities ranged from 5.58 to 91.2 mg g^−1^, primarily influenced by surface area and non‐carbonized organic matter (NOM) content, with physical adsorption dominating in biochar produced at higher temperatures and partitioning in those with higher NOM content. Another study by Zhang et al. investigated biochar from wheat straw, produced with granulated activated carbon using microwave‐assisted pyrolysis.^[^ ^139^ ^]^ The resulting biochar exhibited adsorption capacities of 53.9 mg g^−1^ for benzene, 75.8 mg g^−1^ for toluene, and 63.0 mg g^−1^ for o‐xylene, respectively. The adsorption mechanism for toluene was primarily driven by pore filling; while, the more polar m‐xylene was predominantly adsorbed through partitioning and chemisorption mechanisms. Kaikiti et al. investigated the use of biochar derived from various food sources—pomegranate peels, prickly pear peels, carob, and locust bean gum—for the removal of VOCs such as cresol, dimethyl trisulfide (DMTS), hexane, and benzene.^[^ ^140^ ^]^ Their study revealed that pomegranate‐based biochar, pyrolyzed at 550 °C, achieved complete and rapid removal of these VOCs, attributed to its high specific surface area of 8.3 m^2^ g^−1^. In another experiment, Kaikiti et al. produced biochar by slow pyrolysis of cattle manure at 500 °C and applied it as a thin layer over fresh cattle manure in a glass reactor for 24 h.^[^ ^141^ ^]^ This treatment significantly reduced the emission of VOCs, including sulfur‐based thiols and sulfides, as well as oxygen‐containing VOCs such as alcohols, phenols, and ketones. Notably, emissions of five key VOCs—hexane, DMTS, phenol, p‐cresol, and 2‐methyl‐3‐pentanone—were markedly reduced. Hwang et al. generated nine different biochars from swine manure, poultry litter, coconut shell, and oak, and tested them for the removal of 15 different VOCs emitted from swine manure, including DMDS, DMTS, indolic, phenolic, sulfur compounds, and VFAs.^[^ ^135^ ^]^ They observed that biochar derived from plant biomass exhibited better adsorption for DMDS and DMTS compared to biochar from livestock manure and poultry litter.

Xiang et al. demonstrated that ball‐milled biochar significantly improved VOC removal performance.^[^ ^137^ ^]^ They tested pristine and ball‐milled biochar derived from hickory wood pyrolyzed at 300 °C, 450 °C, and 600 °C. The ball‐milled biochar showed physicochemical improvements, such as enhanced structural properties, increased specific surface area, and higher hydrophilicity and polarity, leading to a 1.3‐ to 13‐fold increase in adsorption compared to pristine biochar. The highest adsorption was observed for acetone (103.4 mg g^−1^), with polar VOCs such as acetone, ethanol, and chloroform primarily influenced by surface area and pore volume. Similarly, Zhuang et al. investigated biochar derived from rice husk, corn stover, and pine wood sawdust pyrolyzed at 300 °C, 500 °C, and 700 °C, revealing a 1.7‐fold enhancement in acetone adsorption capacity (up to 304 mg g^−1^), compared to pristine biochar.^[^ ^142^ ^]^ They concluded that hydrophobic VOCs such as toluene benefit from increased surface area; while, hydrophilic VOCs such as acetone are more influenced by the presence of oxygen‐containing functional groups.

Biochar adsorption of VOCs depends on various factors such as the properties of the adsorbate and adsorbent, the derivative feedstock used for biochar production, the pyrolysis temperature affecting surface area, and the NOM content of biochar. The NOM content also contributes toward the adsorption of specific VOCs such as naphthalene, nitrobenzene, and phthalic acid esters. Adsorption typically occurs through partitioning into the organic phase, particularly in biochars pyrolyzed at lower temperatures with higher NOM content.^[^ ^136^ ^]^ Zhang et al. observed a mixed dual adsorption–partition mechanism in 15 different biochars produced at pyrolysis temperatures of 300 °C, 450 °C, and 600 °C, tested for three common VOCs, including acetone, cyclohexane, and toluene.^[^ ^136^ ^]^ Biochars produced at higher temperatures exhibited lower NOM content and higher surface area, leading to physical adsorption as the dominant mechanism. The correlation analysis between the surface area of biochar and its adsorption capacity revealed a nonlinear relationship with a lower coefficient of determination (*R* ^2^), compared to other carbon‐based materials. This suggests that adsorption involves both physical adsorption and partitioning mechanisms, with the NOM content and surface area in biochar influencing the adsorption capacity. Xiang et al. investigated the mechanisms of various VOCs such as cyclohexane, acetone, chloroform, ethanol, and toluene using ball‐milled biochar derived from hickory wood pyrolyzed at 300 °C, 450 °C, and 600 °C.^[^ ^137^ ^]^ Linear correlation analysis among surface area, adsorption capacity, volatile organic matter, and adsorption rate constant revealed no significant correlations, indicating that the adsorption mechanism followed surface adsorption and partitioning. The biochars demonstrated good reusability after undergoing five adsorption–desorption cycles.

## 4 Biochar as a Potential Material for the Redressal of Radioactive Waste

The presence of radioactive waste in the environment has significant and serious consequences for human health and the natural environment. Radioactive waste can be generated naturally or through anthropogenic activities. The most commonly used radioactive element is uranium, which naturally occurs in trace amounts at 2.5 ppm in the earth's crust.^[^ ^143^ ^]^ The demand for nuclear energy has increased significantly in recent years due to the increasing energy demands and concerns about climate change. This has led to a surge in radioactive waste generation, particularly through nuclear power production, nuclear accidents such as Fukushima and Chernobyl, phosphate fertilizer applications, and mining activities. Naturally, radioactive waste is generated through activities such as rock weathering and volcanic eruptions.^[^ ^144^ ^]^ In addition, research on nuclear energy for medical and industrial purposes has contributed to the increased generation of radioactive waste, leading to elevated levels in soil and heightened chemical and radiological toxicity. Radionuclides with varying half‐lives pose a significant risk due to their radiotoxicity, affecting plant growth and agricultural production and transfer further along the food chain, ultimately impacting harm to human health and diseases such as cancer.^[^ ^145^ ^]^ Isotopes such as U(VI), ^226^Ra, ^137^Cs, and ^90^Sr are commonly found in radioactive waste from nuclear plants, emitting strong radiation. Therefore, it is crucial to remove and effectively treat the generated radioactive waste. Various technologies, including electro‐coagulation, sedimentation, membrane separation, absorptive separation, ion exchange, and vaporization, are employed as remediation methods. Compaction or solidification techniques are also used to reduce waste volume and contain radioactive waste. Immobilization using sorbents is an example of such treatment wherein the radionuclide is separated from the aqueous matrix. This also helps in long‐term storage, preventing or slowing down the release in the environment. Dutta et al. had reported the use of nanocomposite based on SiO_2_ and C from corn cob for U(VI) removal.^[^ ^146^ ^]^ Wang et al. designed ultra‐thin nanosheets of Fe7(PO4)6 with a high specific surface area for high‐capacity U(VI) adsorption.^[^ ^147^ ^]^ However, these methods are complex and cost‐intensive for large‐scale production. Therefore, biochar presents a more feasible alternative due to its affordability, accessibility, and tunability. By modifying biochar properties, such as surface area and pore volume, micro‐structural and selective absorption characteristics can be achieved through physical, biological, and chemical methods. U(VI) adsorption is particularly challenging due to its high solubility compared to U(IV); therefore, efforts have been made toward creating an efficient process for U(VI) adsorption. Surface complexation involves inner‐ and outer‐surface complexation, with the former forming strong bonds for permanent attachment of ions, and the latter is a weak and reversible process usually associated with an electrostatic interaction between the adsorbate and the surface. Immobilization of U ions on biochar surfaces is achieved through the formation of surface complexes via the creation of coordinated bonds between the functional groups attached to the biochar and uranyl ions. Other processes during adsorption include ion exchange, physical adsorption, and reduction processes.^[^ ^148^ ^]^ Guilhen et al. used biochar derived from macauba endocarp at different pyrolytic temperatures; it has been used for U(VI) removal from water.^[^ ^149^ ^]^ Biochars with six different pyrolytic temperatures ranging from 250 °C to 750 °C were explored and found to have varying degrees of U(VI) removal. The authors observed that BC350, a biochar obtained at 350 ° C, was found to ideally be the best among all the six different biochars owing to a good trade‐off between high fixed carbon content and high adsorption capacity. The fixed carbon capacity indicates the stability of the biochar, which is important for long‐term adsorption of radioactive uranium. It is interesting to note that the adsorption of U(VI) was observed to be most efficient at lower pyrolytic temperatures corresponding to a higher H and O content in the biochar having polar organic sites for good adsorbent capacity. It can be inferred that the mechanism of adsorption will follow a chemisorption process with hydroxyl, carboxyl, and carbonyl groups binding to the uranyl ions (UO_2_^2+^). Similarly, to remove U(VI) from the aqueous solution, Ahmed et al. used an oxidized biochar source from rice straw.^[^ ^150^ ^]^ HNO_3_ was used for oxidizing the biochar, which could be chemically enhanced by increasing the presence of groups such as carboxyl, carbonyl, and hydroxyl groups that improve the adsorption capacity of the treated biochar. These were confirmed by the reduced intensity of ─OH/C─O and Fourier Transform Infra‐red (FTIR) stretching vibrations of the [U^−^OU^−^]^2+^ linear structure. This indicated uranyl ion incorporation in the oxidized biochar used for absorption. Further, the oxidized biochar showed good regenerative performance using HCl as an eluent with efficiency higher than 90% even after five cycles. Various factors determine the adsorption of U(VI), among which pH plays a vital role as the surface charge and distribution of adsorbent effects are strongly co‐related. The authors found that U(VI) was best absorbed at a pH of 5.5, and the oxidized biochar had a more negative charge compared to unoxidized biochar, making it more susceptible to absorption of cationic pollutants. Analyzing the adsorption mechanism indicated that the adsorption of U(VI) on the oxidized biochar followed a pseudo‐second‐order reaction model with a slow adsorption process. The intraparticle diffusion model was adopted to understand the migration of particles inside the adsorbent. This indicated that U(VI) adsorption is dominated by chemisorption or strong surface complexation rather than mass transport. Similarly, Philippou et al. also used oxidized biochar for the removal of U‐232 radionuclide from aqueous solutions and observed that the adsorption process is an endothermic and entropy‐driven process that depends upon factors such as temperature, pH, ionic strength, and adsorbent mass.^[^ ^151^ ^]^ Huang et al. recently reported using biochar as a remedial measure for removing uranium contaminants from soil.^[^ ^148^ ^]^ Palansooriya et al. used biochar derived from a feedstock of food waste and wood and activated using KOH for the treatment of water contaminated with ^137^Cs and ^90^Sr.^[^ ^152^ ^]^ The alkaline activation resulted in the enrichment of functional groups such as ─OH, ─NH_2_, and ─COOH that facilitate the adsorption of Cs^+^ and Sr^2+^, thereby forming an outer‐sphere complex. Thus, modified biochar is a promising material that can be used as a cost‐effective means for treating water contaminated with radioactive materials and effectively reducing pollution in both water and soil, which will be beneficial for human health, plants, and soil.

## 5 Machine Learning (ML) and Artificial Intelligence (AI) in Biochar Optimization and Environmental Remediation

**Figure 4:**
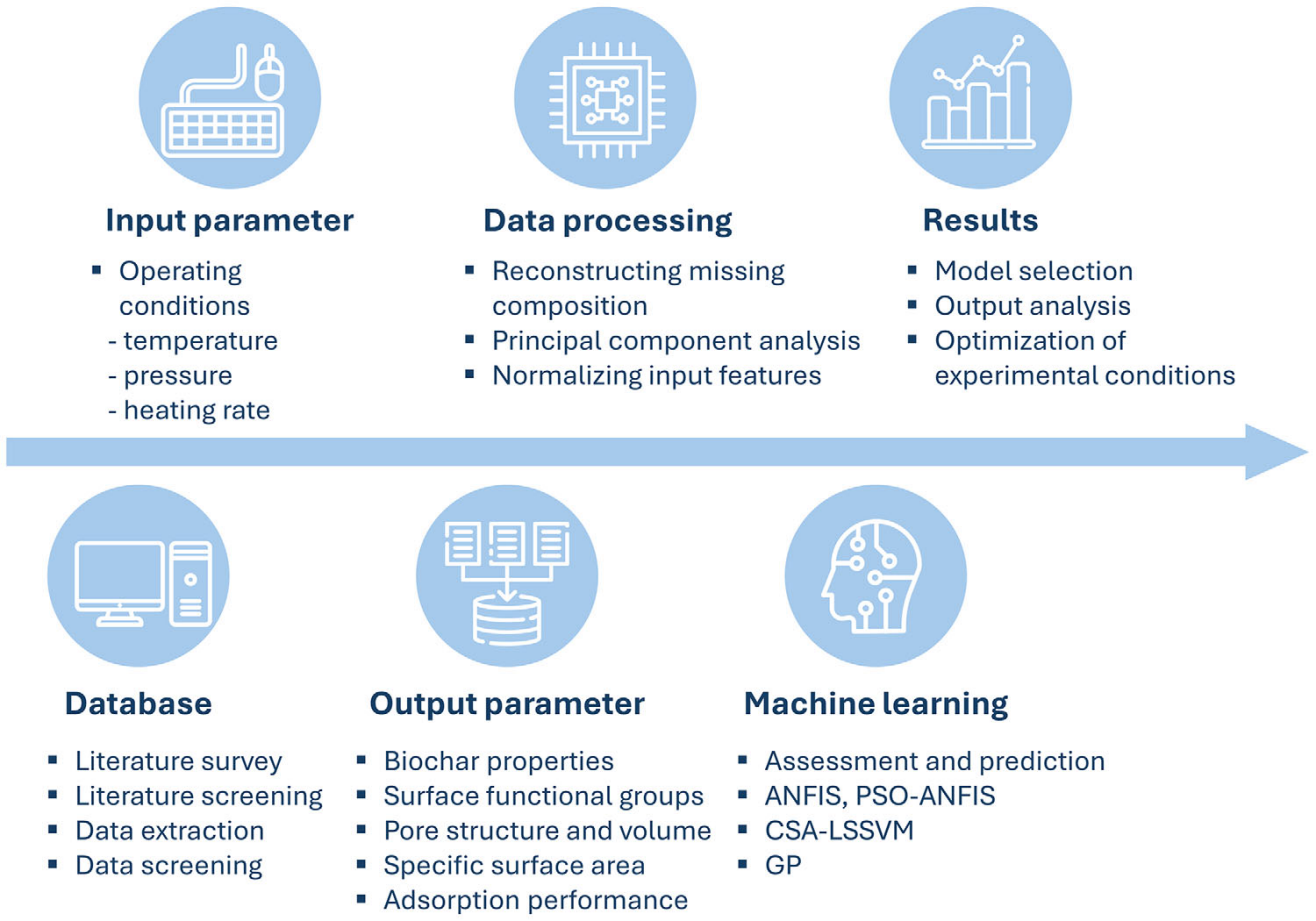
Research methodology for implementing ML in using biochar for water treatment applications.

Advanced techniques such as ML play a crucial role in optimizing biochar by accurately predicting and refining its properties, thereby enhancing its effectiveness in pollutant adsorption. This technology utilizes historical datasets to develop accurate models capable of analyzing nonlinear, complex, and multivariate relationships within the data, significantly reducing the need for lengthy, complex, and costly experimental work. A graphical representation of the ML‐based research methodology is shown in **Figure** **4**. In addition, ML methods have demonstrated superiority over traditional tools such as computational fluid dynamics and thermodynamic equilibrium models in simulating complex chemical processes.^[^ ^153^ ^]^ This data‐driven approach not only streamlines the design process but also minimizes the need for extensive experimental trials, facilitating the development of highly efficient, application‐specific biochar materials for sustainable water treatment.

**Figure 5:**
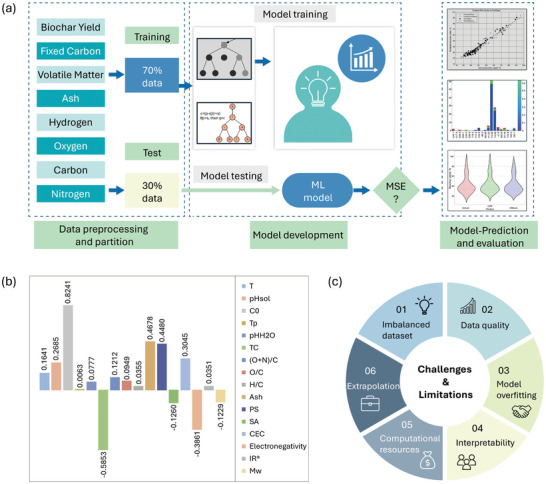
a) Model development of biochar characteristics and yield. Reproduced with permission.^[^ ^156^ ^]^ Copyright 2024, Wiley; b) effect of input parameters on the prediction of biosorption capacity of biochar. Reproduced with permission.^[^ ^155^ ^]^ Copyright 2023, Elsevier; and c) limitations of ML. Reproduced with permission.^[^ ^156^ ^]^ Copyright 2024, Wiley.

ML algorithms are highly effective in accurately predicting and characterizing the properties of biochar, including pore structure, pore volume, surface area, and the presence of functional groups, all of which directly impact its adsorption efficiency for contaminants.^[^ ^154^ ^]^ **Figure** **5**a shows the predictive flowchart for developing a biochar forecasting model. Moreover, ML processes large datasets to identify key features by analyzing complex relationships and patterns, offering valuable insights for the development of biochar‐based adsorbents. For example, Dashti et al. conducted modeling studies on 44 different types of biochar for heavy metal adsorption (including lead, copper, zinc, cadmium, arsenic, and nickel) using data from 353 datasets.^[^ ^155^ ^]^ They employed four ML algorithms: adaptive neuro‐fuzzy inference system (ANFIS), coupled simulated annealing‐least squares support vector machine (CSA‐LSSVM), particle swarm optimization‐ANFIS (PSO‐ANFIS), and genetic programming (GP). These models exhibited excellent predictive performance, with correlation coefficient (*R* ^2^) values of 0.9428, 0.9832, 0.9712, and 0.9750, respectively. CSA‐LSSVM demonstrated the highest accuracy, with mean square error (MSE) and average absolute relative deviation (AARD) values of 0.0020 and 0.36, respectively. The study also highlighted that particle size had a greater impact than the pyrolysis temperature on heavy metal adsorption, demonstrating the potential of ML in effectively designing biochar for enhanced performance. Figure 5b presents a comparative illustration of the effect of various parameters on heavy metal adsorption, with initial concentration having the most significant effect among all parameters. Such studies offer reliable data that are helpful in experimental studies to predict the adsorption capacity of biochar of numerous heavy metal pollutants simultaneously, thereby facilitating remediation from harmful toxic heavy metals in a more efficient and expedited manner.

Traditionally, preparing biochar suited for a particular application involves selecting an appropriate feedstock and optimizing other operational conditions using a strategy known as one‐factor‐at‐a‐time, which includes repetitive experiments. This approach demands significant investments in time, effort, and cost, yielding data with limited reliability. Therefore, there is a critical need to develop a data‐driven process to expedite the process through an intelligent system such as ML. Li et al. adopted two distinct ML models—one for biochar preparation and another for antibiotic adsorption—using the eXtreme Gradient Boosting (XGB) algorithm, achieving *R* ^2^ values ranging from 0.85 to 0.97.^[^ ^87^ ^]^ They also utilized a hybrid ML optimization framework that incorporated particle swarm optimization to design biochar tailored for specific applications, predicting properties such as adsorption capacity, surface area, and pore volume. Their results highlighted that pine wood pyrolyzed at 500—700 °C exhibited the highest adsorption capacity for antibiotics, marking a significant advancement in biochar research. Consequently, an intelligent model was developed to establish a correlation between the parameters of the prepared biochar and its absorption performance, which is particularly crucial for removing targeted pollutants from contaminated water. Pathy et al. similarly utilized XGB ML algorithms to predict the yield and composition of algal biochar, exploring 13 different prototypically important combinations.^[^ ^157^ ^]^ Potnuri et al. employed an ML model based on Leave‐One‐Out using polynomial regression to predict biochar yield, revealing a relationship between sawdust pretreatment and the resulting yield, with an increase in the biochar yield observed with the increasing temperatures during microwave‐assisted pyrolysis.^[^ ^158^ ^]^ Shafizadeh et al. applied four different ML models—NNR, generalized additive model (GAM), support vector regression (SVR), and GPR—to quantitatively and qualitatively analyze the by‐products of hydrothermal liquefaction based on reaction conditions and biomass composition.^[^ ^153^ ^]^ It is interesting to note that such data‐driven models will be highly beneficial in quantitative and qualitative analysis, reducing both time and costs and eliminating the need for expensive trials by streamlining design and analysis processes. A comprehensive overview of various ML studies on biochar is presented in **Table** **1**.

**Table 1:** Comprehensive overview of different studies on biochar using ML and AI.

Biomass source	Type of model	No. of datasets	Operating inputs	Model outputs	Note	Refs.
44 types of biochar	ANFIS, CSA‐LSSVM, PSO‐ANFIS, and GP	353	Characteristics of biochar, biosorption condition, initial concentration, and characteristics of heavy metals	R^2^, MSE, and AARD	Impact of particle size on heavy metal adsorption	[155]
Three feedstocks and Three antibiotics	Hybrid ML model‐based optimization framework via particle swarm optimization	169 and 469 datasets for biochar preparation and Application, respectively	Compositions of different feedstocks and pyrolysis condition	*R* ^2^, specific surface area, total volume, and adsorption capacity	Tailoring biochar for targeted antibiotics	[159]
Lignocellulose biomasses (agricultural residues, forestry residences, and energy crops)	Random forest (RF)	245	Structural component, Elemental compositions, and Particle size of biomass and pyrolysis conditions	Yield and carbon contents of biochar	Improving biochar yield or carbon contents	[160]
Elemental composition and its ratios; and proximate analysis of Algal biomass and pyrolysis condition	eXtreme Gradient Boosting (XGB)	91	H/C, N/C, ash content, pyrolysis temperature, and time	*R* ^2^, algal biochar yield	Prediction of algal biochar yield along with its composition	[157]
Lignocellulosic biomass, herbaceous plants, sewage sludge, and animal manure	Integrated framework of artificial neural networks (ANNs) and metaheuristic algorithms	402	Ultimate analysis, proximate analysis, biomass structural information, and pyrolysis condition	Grey‐wolf optimization, Rao algorithms, sine cosine algorithm, genetic algorithm, and particle swarm optimization (PSO)	Prediction of biochar yield using biomass characteristics and pyrolysis condition	[161]
Agricultural biomass	Multiple linear regression (MLR), decision tree regressor, RF, support vector machine (SVM), and k‐nearest neighbor (KNN),	46	Lignocellulose content and pyrolysis conditions	RF for biochar yield and SSA, feature selection for predicting dependence on operation condition	Accurately estimate biochar yield and SSA	[162]
Sludge	Multilayer perceptron neural network (MLPNN), SVR, and random forest regression (RFR)	244	Sludge ultimate analysis, proximate parameters, and pyrolysis reaction conditions	Biochar yield, atomic Composition, and calorific value	Quantity and quality parameters of sludge pyrolysis products	[154]
Biochar feedstock	Queuing search algorithm (QSA)‐ANN	22 types of biomass feedstock with a total of 44 biochar systems and 353 experiments	Pyrolysis temperature, total carbon, ash, particle size, surface area, CEC, temperature, and pH solution, initial concentration	Root‐mean‐ square error (RMSE) determination coefficient (*R* ^2^) and variance accounted for (VAF)	Predict and improve heavy metal adsorption efficiency onto biochar	[163]
18 different biochars	Bagging, CatBoost, ExtraTrees, HistGradientBoosting, XGBoost, GradientBoosting, DecisionTree, RF, light gradient boosting, and k‐nearest neighbors	3575	24 input variables‐pyrolysis conditions for biochar production, biochar characteristics, compositions, and adsorption experimental conditions	Mean absolute error (MAE) and coefficient of determination (*R*2)	10 different ML models predict adsorption capacity of biochar materials toward the elimination of emerging contaminants	[164]
64 biomasses, including forestry waste, agricultural waste, manure, food waste, algae, grass, sludge, and their mixtures	RF and gradient boosting regression (GBR) models	400	Elemental composition, proximate composition, structural or biochemical composition of biomass, and pyrolysis conditions	Yield, nitrogen content, and specific surface area of biochar	Predict and optimize specific surface area, *N* content, and yield of biochar	[165]
61 lignocellulose biomass, 89 algae, 31 types of biomass feedstocks, and 24 mixed biomass feedstocks	Neural network regression (NNR), GAM, support vector regression (SVR), and Gaussian process regression (GPR)	652	Biomass composition and reaction conditions	MAE and coefficient of determination (*R* ^2^)	Predict the quality and quantity of (by)products of biomass	[153]

ML has a significant potential to transform the use of biochar in environmental remediation, particularly in the removal of heavy metals from wastewater. ML algorithms can precisely identify heavy metals, predict optimal biochar characteristics based on biomass and pyrolysis conditions, and estimate alterations post‐modifications. This predictive ability enables the refinement of biochar production, ensuring its high efficacy for the intended purpose. Besides property prediction, ML can simulate adsorption processes, including reaction mechanisms, kinetics, and thermodynamics, offering a thorough comprehension of biochar interactions with pollutants. This capacity streamlines biochar advancement, diminishing the necessity for extensive experiments and positioning ML as a crucial instrument for propelling biochar research and fostering more sustainable environmental solutions.^[^ ^166^ ^]^ Wang et al. recently emphasized the significance and function of ML algorithms in biochar research.^[^ ^167^ ^]^ They demonstrated how integrating ML into the labor‐ and time‐intensive biochar production and application processes can be more efficiently streamlined using data‐driven ML algorithms alongside mechanism‐based analysis. In a recent review, Nguyen et al. delineated the various challenges and constraints associated with employing ML.^[^ ^156^ ^]^ Key factors influencing predictive forecasts encompass data quality and accessibility, enabling the construction of precise and generalizable models. Some essential parameters are outlined in Figure 5c.

AI—a rapidly advancing technology with significant potential—is increasingly being utilized to optimize wastewater treatment processes by simulating human intelligence. AI techniques such as ANN, RF, and SVM are designed to replicate human thought processes and decision‐making.^[^ ^88^ ^]^ For example, Zheng et al. investigated the removal of six heavy metals—Cu(II), Pb(II), Zn(II), As(III), Cd(II), and Ni(II)—using 44 types of biochar derived from 22 different biomass feedstocks, totaling 353 experimental datasets.^[^ ^163^ ^]^ They applied a QSA to improve the predictive accuracy of ANN models. The QSA–ANN model demonstrated superior accuracy compared to traditional ANN, with a 2.7% and 21.9% improvement in training and testing datasets, respectively, making it a promising tool for practical applications in heavy metal removal from water. Moreno–Perez et al. reported similar work for multi‐component adsorption of Cd, Ni, Zn, and Cu on biochar, employing four neural networks including cascade forward neural network, feed forward back propagation neural network (FFBP), feed forward back propagation neural network with distributed time delay (FFBP‐DTD), and Elman neural network.^[^ ^168^ ^]^ They found that the cascade and FFBP‐DTD models yielded the best results for multi‐metallic adsorption. However, they did not report a breakthrough point in their findings. Therefore, ANN models with reduced modeling errors in the prediction of breakthrough adsorption of multi‐metals need improvement. Further, models that consider and incorporate the physicochemical properties of adsorbates and adsorbents will allow for the development of wide‐scope ANN models.

These predictive models can markedly decrease the necessity for extensive experimental trials, leading to substantial time and cost savings. Moreover, AI can simulate diverse scenarios, enabling the precise adjustment of biochar to target specific contaminants more efficiently. This enhances pollutant removal efficiency and boosts the sustainability and overall effectiveness of water treatment solutions. While AI does not adhere to a rigid formula, its iterative approach—from simple to complex networks—brings predictions closer to actual experimental results, thereby enhancing the accuracy and efficiency of biochar applications in wastewater treatment.^[^ ^169^ ^]^

It can be stated that the use of ML and advanced AI technology can significantly benefit the production and yield of biochar through thorough data analysis and interpretation. This technology has the potential to revolutionize the production and application of biochar by leveraging knowledge to adjust properties based on specific requirements. Identifying key properties and parameters before implementation not only enhances yield but also optimizes resource allocation, reducing waste generation and cutting costs. Incorporating advanced technologies such as AI and ML enables real‐time quality monitoring and timely adjustments to parameters. This approach facilitates informed decision‐making and allows tailored production for specific applications, ultimately reducing costs, time, and waste.

## 6 Summary and Outlook

As global challenges such as water scarcity, environmental pollution, and climate change escalate, the demand for sustainable and effective solutions has become crucial. Biochar, a carbon‐rich material produced from the pyrolysis of biomass, has emerged as a promising tool for addressing these challenges. Its utilization in wastewater treatment, carbon sequestration, soil improvement, and pollution remediation provides a comprehensive approach to environmental management. This review examines the potential of biochar in diverse environmental applications, with a focus on its role in the removal of pollutants from wastewater. The significant findings and prospects of biochar research and its contributions to sustainable development are outlined below.

### 6.1 Optimizing Biochar Production With High Standardization and Quality Control

The biochar production process comprises carefully controlled stages—pretreatment, pyrolysis, and post‐treatment—that can be precisely adjusted to enhance its properties for specific applications. Pretreatment methods, such as chemical activation using acids or alkalis, are pivotal in significantly increasing biochar's surface area, porosity, and functional group density, thereby improving its efficacy in adsorbing pollutants. Subsequent post‐treatment modifications, including physical and biological approaches, further refine these characteristics, enhancing biochar's ability to remove contaminants from water, soil, and air.

Despite the increasing interest in biochar, the field lacks standardized methods for its production and characterization. Establishing protocols that cover feedstock selection, pyrolysis conditions, and post‐treatment processes is crucial to ensure consistent quality and performance across applications. In addition, developing comprehensive guidelines for biochar characterization, focusing on key properties such as surface area, pore structure, functional groups, and stability, will aid result comparison and promote the broader implementation of biochar technologies.

### 6.2 Pollutant Removal Efficacy and Expanding Applications

Biochar's efficacy in wastewater treatment stems from its high surface area, porous structure, and diverse functional groups. These characteristics enable biochar to adsorb a wide range of pollutants, including organic compounds (e.g., dyes and pharmaceuticals), inorganic salts (e.g., nitrates and ammonium), heavy metals (e.g., lead and cadmium), and emerging contaminants, such as microplastics and VOCs. The main mechanisms driving pollutant removal—physical adsorption, ion exchange, electrostatic interactions, complexation, and catalysis—are directly affected by biochar's production conditions and any post‐treatment modifications.

While biochar has shown significant potential in wastewater treatment and pollution remediation, its applications go beyond these areas. Biochar's capacity to improve soil fertility and water retention makes it a valuable tool for sustainable agriculture, especially in regions dealing with water scarcity and soil degradation. In addition, investigating biochar's potential role in carbon capture and storage could significantly contribute to global initiatives to reduce greenhouse gas emissions and address climate change.

### 6.3 Leveraging Advanced Technologies

The integration of ML and AI into biochar research opens new possibilities for optimizing production processes, predicting performance in diverse applications and developing advanced biochar materials with enhanced properties. ML can analyze large datasets to determine the optimal combinations of feedstocks, pyrolysis conditions, and post‐treatment methods for specific environmental challenges. This capability makes ML highly effective in predicting and fine‐tuning biochar properties, significantly reducing the reliance on extensive experimental trials. AI can simulate the interactions of biochar within complex environmental systems, enabling researchers to refine their strategies and achieve superior outcomes in pollutant removal and environmental sustainability.

### 6.4 Managing Environmental and Social Impacts

With the expansion of biochar production and application, addressing potential environmental and social impacts becomes crucial. Although biochar provides substantial environmental advantages, such as carbon sequestration and pollution reduction, prudent management is necessary to avoid unintended outcomes, such as harmful emissions during pyrolysis or excessive depletion of natural resources. In addition, ensuring accessibility and benefits of biochar technologies to communities, particularly in developing regions, is vital for fostering social equity and sustainable development. Future research should focus on optimizing feedstock selection, scaling up production processes, and integrating biochar with other sustainable technologies to maximize its environmental and economic benefits. As the world strives to meet international climate targets and sustainable development goals, biochar stands out as a versatile and scalable solution that can contribute significantly to reducing greenhouse gas emissions and restoring ecosystems. To accelerate the adoption of biochar, it is imperative for policymakers, industry stakeholders, and researchers to collaborate in developing supportive frameworks, funding innovative projects, and promoting public awareness of its benefits. Through continued dedication and global cooperation, biochar has the potential to make a substantial contribution to a more sustainable and resilient future.

In conclusion, biochar offers a promising solution to key environmental challenges, including pollutant removal, carbon sequestration, and soil remediation. By leveraging advanced technologies such as machine learning and artificial intelligence, biochar production and application can be optimized for greater efficiency and scalability. To fully harness its potential, standardized production methods and further exploration of its diverse applications are essential. With continued research and collaboration, biochar can play a significant role in advancing global sustainability efforts.

## Conflict of Interest

The authors declare no conflict of interest.
